# Proteomic Profiling of Thyroid Papillary Carcinoma

**DOI:** 10.1155/2012/815079

**Published:** 2012-02-12

**Authors:** Yoshiyuki Ban, Gou Yamamoto, Michiya Takada, Shigeo Hayashi, Yoshio Ban, Kazuo Shimizu, Haruki Akasu, Takehito Igarashi, Yasuhiko Bando, Tetsuhiko Tachikawa, Tsutomu Hirano

**Affiliations:** ^1^Division of Diabetes, Metabolism and Endocrinology, Department of Medicine, Showa University School of Medicine, 1-5-8 Hatanodai, Shinagawa-ku, Tokyo 142-8666, Japan; ^2^Department of Oral Pathology and Diagnosis, School of Dentistry, Showa University, 1-5-8 Hatanodai, Shinagawa-ku, Tokyo 142-8555, Japan; ^3^Ban Thyroid Clinic, 2-11-16 Jiyugaoka, Megro-ku, Tokyo 152-0035, Japan; ^4^Division of Endocrine Surgery, Department of Surgery, Nippon Medical School, 1-1-5 Sendagi, Bunkyo-ku, Tokyo 113-8602, Japan; ^5^Biosys Technologies, Inc., 2-13-18 Nakane, Meguro-ku, Tokyo 152-0031, Japan; ^6^Comprehensive Research Center of Oral Cancer, Showa University, 1-5-8 Hatanodai, Shinagawa-ku, Tokyo 142-8555, Japan

## Abstract

Papillary thyroid carcinoma (PTC) is the most common endocrine malignancy. We performed shotgun liquid chromatography (LC)/tandem mass spectrometry (MS/MS) analysis on pooled protein extracts from patients with PTC and compared the results with those from normal thyroid tissue validated by real-time (RT) PCR and immunohistochemistry (IHC). We detected 524 types of protein in PTC and 432 in normal thyroid gland. Among these proteins, 145 were specific to PTC and 53 were specific to normal thyroid gland. We have also identified two important new markers, nephronectin (NPNT) and malectin (MLEC). Reproducibility was confirmed with several known markers, but the one of two new candidate markers such as MLEC did not show large variations in expression levels. Furthermore, IHC confirmed the overexpression of both those markers in PTCs compared with normal surrounding tissues. Our protein data suggest that NPNT and MLEC could be a characteristic marker for PTC.

## 1. Introduction

Papillary thyroid carcinoma (PTC) is the most common form of the follicular-cell-derived carcinomas and comprises three quarters of all newly diagnosed thyroid cancers [1]. PTC is derived from the follicular cells. These cells tend to concentrate iodine and secrete thyroglobulin. As a result, surveillance and detection of recurrence can be relatively straightforward. The prognosis for PTC is usually excellent (reviewed in [2]).

Over the past 10–15 years, several candidate genes have been studied in the development of different types of thyroid cancer (e.g., TSH receptor, RET/PTC, Ras, BRAF, and p53) [3–5]. These genes have been evaluated in thyroid cancer based on what was known or other cancers or based on what was expected from normal cell signaling (protooncogenes). Recent studies using gene array technology have attempted to use a hypothesis-generating approach to understand thyroid neoplasms [6–8], but these studies rely on mRNA differences that may not be related to significant biologic processes. mRNA differences do not necessarily reflect differences at the protein level, and these RNA-based studies fail to identify protein variants and posttranslational modifications that affect the tumor biology.

Proteomics is the name given to a set of analytical strategies that can simultaneously identify and quantify thousands of protein components in a biological sample [9]. There is, however, no single approach that optimally meets this demanding objective; instead, several complementary technologies have emerged, each offering distinctive strengths and weaknesses. These approaches are now facilitating new biomarker discoveries in many areas of medicine. Studies of the thyrocyte and thyroid cancer cell proteome are in their infancy compared with studies of the genome and transcriptome [9].

We employed nanoflow liquid chromatography and mass spectrometry, followed by protein identification by tandem mass spectrometry (LC/MS/MS) to gain a better understanding of thyroid cancer and the unique alterations that are characteristic of PTC. This technique provides an accurate quantitative comparison of two groups of samples, allowing the identification of proteins whose levels differ significantly between the two conditions. Using this approach, we have identified novel differentially expressed proteins that may provide insights into diagnosis, prognosis, and therapeutic targets for patients with thyroid neoplasms, as well as into the underlying pathophysiology of thyroid tumor development and progression.

## 2. Materials and Methods

### 2.1. Ethics Statement

The research protocol was approved by the Ethic Committee of the Showa University Hospital, and each subject signed the informed consent form approved by the Institutional Review Board at the Showa University Hospital.

### 2.2. Thyroid Tissue Samples

Tumor and matched normal thyroid tissue was collected from four patients undergoing surgery for PTC. Specimens (100–500 mg) from each patient, verified by histopathology, were snap frozen after confirmation of tissue type. Tissue samples were collected from women aged 28–49 years with no evidence of chronic lymphocytic thyroiditis in an attempt to minimize differences due to gender, menopausal status, and autoimmune thyroid disease. Unaffected (normal) thyroid tissue was defined as tissue adjacent to the site of the lesion with no histologic signs of abnormal pathology. Normal tissue was collected from each patient undergoing surgery for PTC so that the biologic variability in protein expression in a region proximate to pathology could be assessed. All cases were analyzed by real-time (RT) PCR analysis and immunohistochemistry (IHC), while LC/MS/MS was applied on 3 out of 4 cases. The clinical details of patients are summarized in Table 1.

### 2.3. Peptide Extraction for LC/MS/MS Analysis

Proteins from samples for LC/MS/MS were extracted using 10 *μ*L of 8 M Urea solution with an ultrasonic homogenizer. After homogenization, 90 *μ*L of 90% 100 mM Ammonium Bicarbonate Buffer (ABB: pH 8.0)/10% Acetonitrile were added, followed by addition of 4 *μ*L of 100 mM dithiothreitol in ABB. Samples were incubated at 37°C for 60 min and were cooled at room temperature. Next, 10 *μ*L of 100 mM iodoacetamide in ABB was added, and samples were incubated at 37°C for 30 min in the dark. Finally, proteins in the samples were digested with trypsin (15–18 units) by overnight incubation at 37°C. After extraction, all samples were stored at −20°C until LC/MS/MS analysis.

### 2.4. Shotgun Liquid Chromatography (LC)/Tandem Mass Spectrometry (MS/MS)

Peptide-mixture samples processed from each tissue were used for nanoflow reverse phase liquid chromatography followed by tandem MS, using an LTQ linear ion-trap mass spectrometer (Thermo Fischer, San Jose, CA). The capillary reverse phase HPLC-MS/MS system (ZAPLOUS System; AMR, Tokyo, Japan) was composed of a Paradigm MS4 dual solvent delivery system (Michrom BioResources, Auburn, CA), an HTC PAL autosampler (CTC Analytics, Zwingen, Switzerland), and Finnigan LTQ linear ion-trap mass spectrometers (ITMS; Thermo Fischer, San Jose, CA) equipped with an XYZ nanoelectrospray ionization (NSI) source (AMR, Tokyo, Japan).

All samples were evaporated, and peptides were redissolved with MS-grade water containing 0.1% trifluoroacetic acid and 2% acetonitrile (solvent A). Aliquots of 10 *μ*L (equivalent to 1 *μ*g of protein) were automatically injected into a peptide Cap-trap column (Michrom BioResources) attached to an injector valve for desalinating and concentrating peptides. After washing the trap with solvent A, peptides were loaded onto a separation capillary reverse phase column (Mono Cap 150 × 0.2 mm; GL Sciences, Tokyo, Japan) by switching the valve. The eluents used were: A, 98% H_2_O/2% acetonitrile/0.1% formic acid; B, 10% H_2_O/90% acetonitrile/0.1% formic acid. The column was developed at a flow rate of approximately 1 *μ*L/min with the concentration gradient of acetonitrile, as follows: first, from 5% B to 55% B in 100 min, then from 55% B to 95% B in 1 min, maintenance at 95% B for 9 min, then from 95% B to 5% B in 3 min, and finally reequilibration with 5% B for 15 min.

Effluents were introduced into the mass spectrometer via the NSI interface, which had a separation column outlet connected directly with an NSI needle (150-*μ*m OD/20-*μ*m ID FortisTip; OmniSeparo-TJ, Hyogo, Japan). ESI voltage was 2.0 kV, and the transfer capillary of the LTQ inlet was heated at 200°C. No sheath or auxiliary gas was used. The mass spectrometer was operated in a z range of 450–1800 in a data-dependent acquisition mode, in which detecting the most abundant ions at a retention time automatically acquires MS/MS scans for those ions under the control of Xcalibur software (Thermo Fischer) with an isolation width of *m/z* 2.0 and a collisional activation amplitude of 35%. Full-scan MS used enhanced/centroid mode, and sequential MS/MS used normal/centroid mode, with dynamic exclusion capability, which allows sequential acquisition of MS/MS of abundant ions in the order of their intensities with an exclusion duration of 1.0 min and exclusion mass widths of −1 and +2 Da. The trapping time was 50 ms using auto gain control.

All MS/MS spectral data were searched against the SwissProt 57.3 Homo sapiens database (468,851 entries) using Mascot (version_2.1.04, Matrix Science, London, UK), in which the peptide and fragment mass tolerances were 2.0 Da and 0.8 Da, respectively. For variable peptide modifications, methionine oxidation and carbamidomethyl (Cys) were taken into account. A *P* value less than 0.05 was considered to indicate a statistically significant difference, and the expected score cut-off was 0.05. Reported results were obtained from triplicate LC-MS runs for each sample with all peptide hits included. Unique peptides and proteins were determined by following proteomics guidelines. Relative abundances of identified proteins were also obtained using the normalized spectral abundance factor (NSAF) introduced by Kawamura et al. and Zybailov et al. [10, 11].

### 2.5. Laser Microdissection and Semiquantitative Real-Time PCR

Tumor cells of PTC and follicular epithelium of normal thyroid tissue (periphery of PTC area) were collected from frozen sections by laser microdissection. Total RNA was extracted from each population of laser-microdissected cells using an RNeasy Plus Micro kit (QIAGEN, Valencia, CA) according to the manufacturer's instructions. Reverse transcription was carried out in 20-*μ*L volumes using a High Capacity RNA to cDNA MasterMix (Applied Biosystems, Carlsbad, CA).

PCR was performed using an ABI PRISM 7500 Sequence Detection System (Applied Biosystems), and analysis was carried out using the sequence detection software supplied with the instrument. Each reaction mixture contained 10 *μ*L of TaqMan Gene Expression Master Mix (Applied Biosystems), 1 *μ*L of TaqMan Gene Expression Assay primer (Applied Biosystems), and 2 *μ*L of template cDNA supplemented with RNase-free water to a final volume of 20 *μ*L. Primers were positioned to span exon-intron boundaries, reducing the risk of detecting genomic DNA. Each PCR consisted of 10 min at 95°C for enzyme activation, followed by 50 cycles of denaturation at 95°C for 15 s and annealing/extension at 60°C for 1 min. Negative control (RNA with no reverse transcription) was included to control for DNA contamination. The housekeeping gene glyceradehyde-3-phosphate dehydrogenase (GAPDH: Assay ID Hs99999905_m1) was used as an endogenous control. Expression values of nephronectin (NPNT) (Assay ID Hs00405900_m1) and malectin (MLEC) (Assay ID Hs00207082_m1) were normalized against the GAPDH values for each sample. Each sample was run in triplicate, and the means were used in semiquantitative analysis.

### 2.6. Immunohistochemical Study

Immunohistochemical analysis using the DAKO EnVision system (DAKO, Carpinteria, CA) was performed. Each sample was studied using H-E staining and immunohistochemical staining. Frozen sections were fixed for 30 min in 4% paraformaldehyde and were washed with phosphate-buffered solution (PBS, pH 7.4) three times for 3 min each. Blocking reagent (DAKO) was then applied at room temperature for 10 min in order to prevent nonspecific binding of antibodies, and sections were washed in PBS three times for 3 min each. In this study, anti-MLEC rabbit polyclonal (Sigma-Aldrich Inc., St. Louis, MO) and Anti-POEM/NPNT rabbit polyclonal (Trans Genic Inc., Kobe, Japan) antibodies were used. Antibodies were diluted 200× and were dropped onto sections for 60 min at room temperature. Sections were incubated with polymer reagent (DAKO EnVision) for 30 min. After washing with PBS, sections were incubated with 3,3′-diaminobenzidine tetrahydrochloride (DAKO) for 1-2 min. Finally, sections were washed with distilled water, counterstained with hematoxylin for 1 min, washed with tap water and ethanol, and covered with cover slips.

### 2.7. Statistical Analysis

All values are expressed as means ± SD. The statistical significance of differences between groups was analyzed by unpaired Student's *t*-test. A *P* value of less than 0.05 was considered to be statistically significant.

## 3. Results

### 3.1. Protein Identification by LC/MS/MS

**Figure 1:**
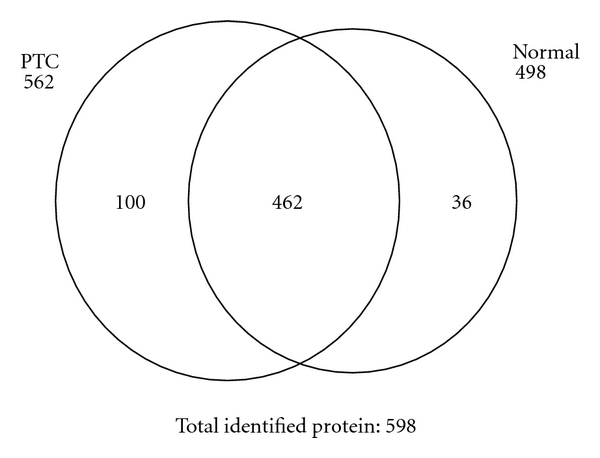
Venn diagram of identified proteins. In PTC, 562 proteins were identified, and 498 were identified in normal thyroid tissue, with 462 proteins being common to both PTC and normal thyroid tissue. The total number of identified proteins was 598 (Normal; normal thyroid tissue; PTC; Papillary thyroid carcinoma).

**Figure 2:**
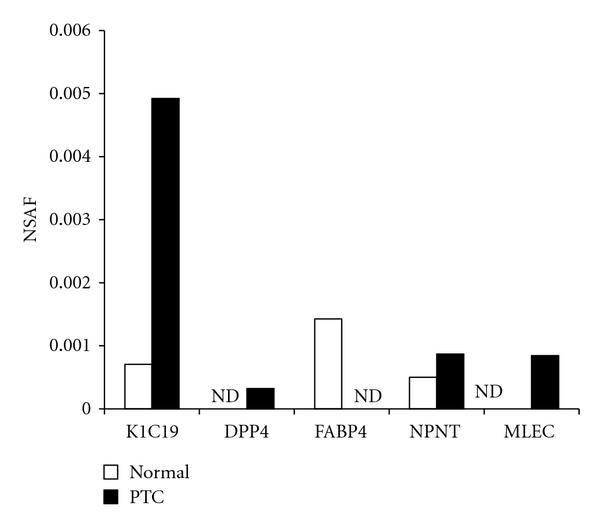
Comparison of NSAF in the identified proteins in PTC tissues (*n* = 3) with NSAF in normal thyroid tissues (*n* = 3). Similar to previous reports, Keratin 19 (K1C19) and Dipeptidyl peptidase 4 (DPP4) were strongly expressed in PTC, and Fatty acid-binding protein 4 (FABP4) was strongly expressed in normal thyroid tissues. NPNT and MLEC were discovered as strongly expressed candidate markers in PTC (Normal: normal thyroid tissue, PTC: Papillary thyroid carcinoma, ND: not detectable).

Protein identification results for triplicate injections were merged and proteins detected twice or more were studied. In PTC and normal thyroid tissue, the numbers of proteins were 562 and 498, respectively, and 462 proteins were common to both. Thus, 100 proteins were unique to PTC, and 36 to normal thyroid tissue (Figure 1). Among the identified proteins in both tissue types, several known markers and two new markers (NPNT and MLEC), with large numbers of NSAF, are shown in Figure 2. Previous studies indicated that Keratin 19 (K1C19) and Dipeptidyl peptidase 4 (DPP4) were strongly expressed in PTC, and Fatty acid-binding protein 4 (FABP4) was strongly expressed in normal thyroid tissues [12]. These observations suggest that our results reflect characteristics unique to each type of membrane, as well as common features between the three. As shown in Figure 2, two new candidate markers, NPNT and MLEC, were strongly expressed in PTC.

### 3.2. Gene Expression of NPNT and MLEC in PTC and Normal Thyroid Tissues

**Figure 3:**
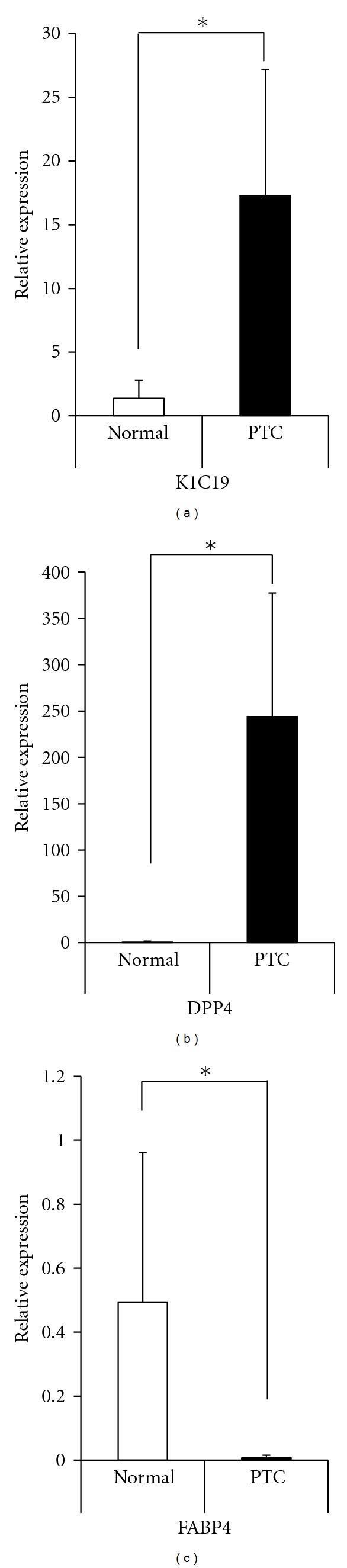
Gene expression of previously known markers (K1C19, DPP4, and FABP4) in normal thyroid tissue and PTC. Gene expression levels of K1C19 and DPP4 were significantly higher in PTC when compared with normal thyroid tissue. In contrast, FABP4 expression was lower (Normal: normal thyroid tissue, PTC: Papillary thyroid carcinoma, *P < 0.05, Bar: 1 SD).

**Figure 4:**
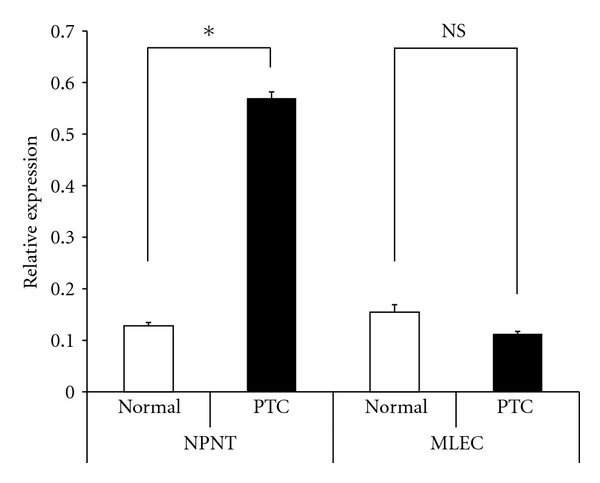
Gene expression of new candidate markers (NPNT and MLEC) in normal thyroid tissue and PTC. Gene expression of NPNT was significantly higher in PTC when compared with normal thyroid tissue. However, expression of MLEC showed no significant differences (Normal: normal thyroid tissue, PTC: Papillary thyroid carcinoma, *P < 0.05, NS: not significant, Bar: 1 SD).

In order to confirm the validity of the LC/MS/MS results, semiquantitative real-time PCR was performed with a set of human-specific primers and template cDNA generated by reverse transcription. Similarly to the LC/MS/MS results and previous reports, the levels of K1C19 and DPP4 expression in the PTC were significantly higher than in normal thyroid tissue, while FABP4 levels were lower (Figure 3). With regard to new candidate markers, although the levels of NPNT expression were significantly higher in PTC, there were no significant differences in MLEC expression (Figure 4).

### 3.3. Immunohistochemical Analysis of NPNT and MLEC in PTC and Normal Thyroid Tissues

**Figure 5:**
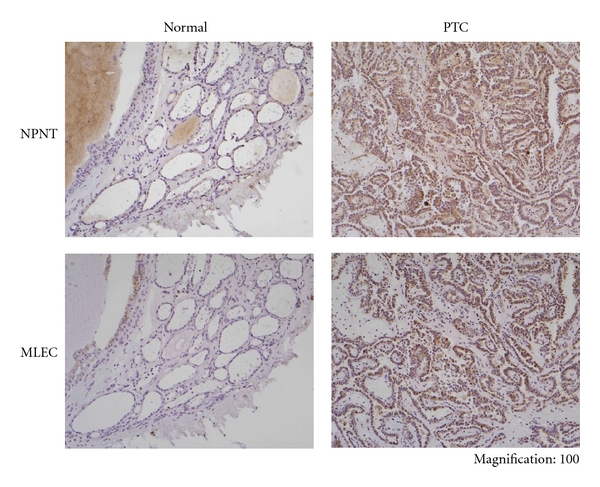
Immunohistochemical localization of NPNT and MLEC in normal thyroid tissue and PTC. Only weak staining of NPNT and MLEC were observed in follicular epithelium of normal thyroid tissue (surrounding tumor). In PTC, strong staining was seen with both antibodies (Normal: normal thyroid tissue, PTC: Papillary thyroid carcinoma).

The distribution of NPNT and MLEC protein expression in normal thyroid tissue and PTC was examined by immunohistochemical analysis. Few positive reactions for NPNT and MLEC were observed in the follicular epithelium (surrounding tumor); on the other hand, carcinoma cells were strongly stained (Figure 5). Particularly for MLEC, no significant differences in gene expression were observed between normal thyroid tissue and PTC; nevertheless, the protein localization was clearly different.

## 4. Discussion

MS-based proteome analysis of frozen tissue sections has identified thousands of unique proteins in various histological tissue samples. The results presented herein demonstrate that a protein solubilization methodology allows global proteomic investigation of tissues. In the present study using our proteome platform, we identified two new markers for PTC, NPNT and MLEC, as well as several known markers, such as KRT19 and DPP4, with significantly increased expression in tissue samples from PTC.

PreOsteoblast EGF-like repeat protein with MAM domain (NPNT) was originally identified in developing mouse organs, particularly at epithelial-mesenchymal interfaces in tissues undergoing morphogenesis [13, 14]. The protein was determined to be associated with cells or with the extracellular matrix but was not found in culture medium, leading to the hypothesis that NPNT is a matrix protein binding to the cell surface. This agrees with an arg-gly-asp binding domain and an integrin binding site found in the amino acid sequence, as well as with a study showing that integrin *α*-8 *β*-1 binds to NPNT [14]. Binding to other integrin molecules has not specifically been observed to date. In a recent study by Eckhardt et al., NPNT was first suggested to be involved in cancer [15]. They applied a genomic analysis to cell lines derived from a spontaneous breast cancer model in mice, and NPNT expression was shown to be related to metastasis. In breast cancer, strong NPNT expression was found in the tumor epithelium of high-metastasis tumors.

MLEC is a novel carbohydrate-binding protein in the endoplasmic reticulum (ER) and is a candidate player in the early steps of protein N-glycosylation [16]. The recent discovery of MLEC, an ER-resident protein that binds oligosaccharides displaying terminal glucose residues with a strong preference for di-glycosylated residues in vitro [16–18], led to speculation on the possible involvement of this lectin in the calnexin chaperone system and in glycoprotein quality control in the mammalian ER [19, 20]. Recently, Galli et al. [21] showed that MLEC is an ER stress-induced type I membrane protein that is associated with newly synthesized glycoproteins in living cells. Analysis of the influenza virus HA revealed that calnexin and MLEC have distinct kinetics in association with newly synthesized polypeptides and that MLEC is preferentially associated with misfolded HA conformers [21]. Changes in the intraluminal levels in MLEC did not affect the function of the calnexin chaperone system or the maturation of HA, an obligate calnexin substrate [21]. It is, therefore, unlikely that MLEC participates in the calnexin chaperone system [21].

There are three main limitations to the present study. The first involves the intrinsic limitations of LC/MS in the analysis of complex protein mixtures. Proteins of very high or low molecular mass are frequently eliminated from analysis by the electrophoresis procedure itself. Conversely, even after depletion of very abundant proteins, both detection by LC and identification by MS are limited by the abundance of the proteins in the mixture, and minor components will always escape analysis. Therefore, candidates proteins at picogram levels cannot be identified. The second limitation pertains to the rigorous selection of membrane samples, which does not allow the inclusion of an ample set of samples and may consequently impede the consideration of other candidate proteins existing in the tissues of PTC patients. However, although the number of samples included in this study was low, the selection process enabled us to minimize the dispersion of the measurements in each group. The observed differences by LC/MS were further validated by RT-PCR analysis of samples from different patients, which confirmed the observed differences with good quantitative agreement. Therefore, although additional differentially expressed proteins could probably be identified by analyzing a larger set of samples, those reported herein would likely also be found. Another limitation is that our data does not demonstrate whether NPNT and MLEC cause PTC, or are simply over-expressed due to tissue proliferation in PTC. Moreover, apparently contrasting data (proteomic analysis and IHC on one side, gene expression on the other side) for malectin are shown, while for nephronectin they are all concordant. This discrepancy may be due to protein variants or posttranslational modifications that affect the tumor biology. Further studies are necessary in order to elucidate whether NPNT and malectin play a role in the etiology of PTC.

## 5. Conclusions

Recent advances in proteomic technologies are increasingly being applied to the study of clinical samples in the search for diagnostic biomarkers and therapeutic targets. Herein, we demonstrated that the powerful combination of LC/MS/MS and frozen tissue samples, for which matching clinicopathological information is available, is beginning to show promise as a research tool. Using this method, we may thus be able to identify new markers that cannot be distinguished on gene expression analysis. Although the number of samples included in this study was low, these data suggest that NPNT and MLEC are characteristic markers and therapeutic targets for PTC.

## Figures and Tables

**Table 1:** Clinical details of analyzed tissue samples.

No.	Age (yr)	Gender	Possible PTC-subtype	Proteomics	RT-PCR	IHC
1	49	F	Common type	+	+	+
2	41	F	Common type	+	+	+
3	28	F	Common type	+	+	+
4	43	F	Common type	−	+	+

F: Female; PTC: papillary thyroid cancer; IHC: Immunohistochemistry; −: not tested.

## Abbreviations

PTC: Papillary thyroid carcinoma
LC/MS/MS: Shotgun liquid chromatography/tandem mass spectrometry
RT-PCR: Real-time PCR
IHC: Immunohistochemistry
NPNT: Nephronectin
MLEC: Malectin
NSAF: Normalized spectral abundance factor
GAPDH: Glyceradehyde-3-phosphate dehydrogenase.
